# Association of oral dysbiosis with oral cancer development

**DOI:** 10.3892/ol.2020.11441

**Published:** 2020-03-03

**Authors:** Giusy Rita Maria La Rosa, Giuseppe Gattuso, Eugenio Pedullà, Ernesto Rapisarda, Daria Nicolosi, Mario Salmeri

**Affiliations:** 1 Department of General Surgery and Surgical-Medical Specialties, University of Catania, I-95125 Catania, Italy; 2 Department of Biomedical and Biotechnological Sciences, International PhD Program in Basic and Applied Biomedical Sciences, University of Catania, I-95123 Catania, Italy; 3 Department of Biomedical and Biotechnological Sciences, University of Catania, I-95123 Catania, Italy; 4 Department of Biomedical and Biotechnological Sciences, Research Center for Prevention, Diagnosis and Treatment of Cancer, University of Catania, I-95123 Catania, Italy

**Keywords:** microbiota, microbiome, oral cancer, carcinogenesis, chronic inflammation, dysbiosis, oral squamous cell carcinoma, probiotics

## Abstract

Oral squamous cell carcinoma (OSCC) is the leading cause of mortality for oral cancer. Numerous risk factors mainly related to unhealthy habits and responsible for chronic inflammation and infections have been recognized as predisposing factors for oral carcinogenesis. Recently, even microbiota alterations have been associated with the development of human cancers. In particular, some specific bacterial strains have been recognized and strongly associated with oral cancer development (*Capnocytophaga gingivalis, Fusobacterium spp., Streptococcus spp., Peptostreptococcus spp., Porphyromonas gingivalis* and *Prevotella spp*.). Several hypotheses have been proposed to explain how the oral microbiota could be involved in cancer pathogenesis by mainly paying attention to chronic inflammation, microbial synthesis of cancerogenic substances, and alteration of epithelial barrier integrity. Based on knowledge of the carcinogenic effects of dysbiosis, it was recently suggested that probiotics may have anti-tumoral activity. Nevertheless, few data exist with regard to probiotic effects on oral cancer. On this basis, the association between the development of oral cancer and oral dysbiosis is discussed focusing attention on the potential benefits of probiotics administration in cancer prevention.

## 1. Introduction

Oral squamous cell carcinoma (OSCC) is the leading cause of death among all oral cancers. This tumor originates from the oral mucosa and accounted for over 350,000 new diagnoses and more than 175,000 recorded deaths worldwide in 2018 (1). It was widely demonstrated that the development of tumors, including that of OSCC, is sustained by several risk factors and predisposing conditions such as fibers, chemicals, pesticides and heavy metals able to induce pro-oncogenic genetic and epigenetic alterations (2–6). Other factors, including oral injuries, inflammatory diseases, infections, and bacterial dysbiosis are now recognized as risk factors for cancer development (7–10).

Regarding OSCC, it was demonstrated that its development is strictly influenced by host-related and lifestyle factors mainly represented by smoking, alcohol abuse, tobacco and tobacco-derivate chewing and oral virus infections (HPV) (11,12). In addition, it was demonstrated that the combination of smoke, alcohol drinking and poor oral hygiene increase the risk of oral cancer onset due to chronic inflammation and infection which constitute the principal factors involved in cancer pathogenesis, influencing the resident microbiota that are involved in the homeostasis of the oral environment (13–17). Importantly, a precise distinction must be made between microbiota and microbiome: the former is comprised of all the bacteria species hosted within the oral cavity, while with the latter term is used to define the collective genomes of microorganisms inhabiting the oral mucosa (18–20).

Oral microbiota changes are able to modulate the connection between oral bacteria and humans causing diseases (21,22). Oral microbiota seems to influence OSCC through the carcinogenetic modulation of cell metabolism (such as modifying the concentration of nutrients and vitamins), thereby promoting the production of different cytokines known to be involved in several pathological conditions (23–27).

Over 600 bacterial species constitute the oral microbiota. However, the majority of these species are uncultivated (19). The availability of new sequencing technology allowed the identification of bacterial communities that harbor the oral cavity and that are involved in human health (28) (Table I).

Researchers have investigated the possible association between microbes and the alteration of physiological conditions. In this context, it was demonstrated that gut microbiota predisposes individuals for the development of different diseases including celiac disease, neurological disorders and blood pressure alterations (29–31). All these pathological conditions are involved in the development of severe health conditions mainly represented by neurovascular disorders, chronic degenerative diseases, and cancer (32–46). Among all cancers, oral cancer is particularly related with oral and gut microbiota composition as widely demonstrated. Among the different bacteria strongly associated with OSCC, *Fusobacterium nucleatum, Porphyromonas gingivalis*, and *Prevotella intermedia* are considered the most represented bacteria types (47–51). Moreover, other bacterial genera, such as *Actinomyces, Clostridium, Enterobacteriaceae, Fusobacterium, Haemophilus, Porphyromonas, Prevotella, Streptococcus spp*. and *Veillonella* are associated with pre-cancerous lesions and oral cancer (52). According to other studies, a high bacterial load of *Prevotella melaninogenica, Streptococcus mitis* and *Capnocytophaga gingivalis* are identifiable in saliva samples of OSCC patients (Table II) (50,51).

Although the association between some species and oral cancer was already established, the complexity of the relationship occurring between cancer and oral microbiota remains unexplained and cannot be limited to the evaluation of a single pathogen (53). Moreover, there are no concordant analytical protocols for the analysis of oral microbiota and microbiome. Therefore, it is difficult to establish oral cavity-associated microbial patterns in cancer patients and healthy subjects. Consequently, there is a lack of novel microbial biomarkers for the early identification of oral carcinoma (54,55).

Previous findings demonstrated that a strong association between oral microbiota and oral cancer exists. Starting from this supposition, a number of studies focused on the prevention of neoplastic transformation and retardation of cancer progression by modulating the carcinogenic or protective microbiome. In this context, probiotics administration was recently considered a good cancer preventive strategy due to the immunological effects (8,9,25). The beneficial effects of lactic ferments and probiotics were identified in the 19th century by Dr Ilya Metchnikoff. Nevertheless, only in recent years have these products been widely used for the treatment of several diseases (56,57). Numerous studies have shown the potential positive effects of probiotics on cancer through several mechanisms that include immune modulation, the prevention of pathogen infections, inflammatory modulation, reduced cancer formation and metastatic process (9,25). To the best of our knowledge, few data have been generated about the effects of probiotics in oral cancer development. Of note, the results of a previous study demonstrated that the administration of *Lactobacillus rhamnosus* GG (LGG) was able to increase the effects of geniposide, an anticancer molecule tested on human oral squamous carcinoma cells (HSC-3), demonstrating the beneficial role of LGG as potential adjuvant of geniposide treatment (58).

The aim of this review was to describe the scientific evidence collected during the years pertaining to oral microbiota and neoplastic transformation with special attention for OSCC. Finally, a brief overview on the anti-tumoral effect of probiotics and their applications in oral cancer was reported.

## 2. Impact of oral health dysregulation on oral cancer development

Observational studies have shown a link among oral cancer and infrequent tooth brushing, infrequent dental visits and loss of or missing teeth (59–62). These findings, however, pertain only to non-smokers and non-drinkers (13–14). Another study revealed that periodontal illnesses are correlated with an increased risk for oral tumors (63). Furthermore, research performed on 51 tongue cancer patients and 54 normal controls cases revealed that chronic periodontal inflammation is a cancer risk factor (64). In addition, periodontitis patients showed an increased risk for OSCC compared to healthy controls (65). Another observational study conducted on a wide cohort of individuals in the USA investigated the use of dental care and oral cancer risk. The analysis of covariates and dental care appointments demonstrated that individuals with a dental appointment during the past 12 months had a lower (62%) oral cancer risk compared with subjects that had not used dental care procedures in the past year (66).

According to these results, the research group of Börnigen *et al* (67) analyzed the role of oral microbiome and its composition by analyzing the biological samples of 121 oral cancer patients and 242 healthy controls matched for age and sex. The multivariate analyses highlighted significant variations of the oral microbiome in subjects with poor dental hygiene, in smokers, and oral cancer patients. In particular, although the microbiome alterations in cancer patients were significant, the alterations were more evident after tooth loss. Therefore, findings of that study showed that both oral microbiome alterations and tooth loss constitute important risk factors for oral cancer development due to the molecular and microenvironmental changes occurring in the oral cavity after these events (67).

## 3. Possible mechanisms of carcinogenesis induced by dysbiosis

The association between gut microbiota and gastric cancer is well known (68). However, the association between oral cancer and oral dysbiosis is not fully understood (69). Different mechanisms of action to elucidate the oral microbiota influence on cancer pathogenesis, including bacterial stimulation of chronic inflammation have been reported. This process causes the production of inflammatory mediators that can cause or facilitate mutagenesis, uncontrolled cell proliferation, angiogenesis and cell degeneration responsible for neurodegenerative disorders and cancer (70–72). In addition, bacteria are able to modulate cell proliferation through activation of the nuclear factor κB (NF-κB) and the inhibition of cell apoptosis promoting or inhibiting the development of several cancer types (73–75).

Moreover, Pang *et al* specified that the integration of virus oncogenes into host genomes or the alteration of epithelial barrier integrity could promote genome instability and favor irreversible cellular damage (76). In this context, it is noteworthy that the complex interaction among microbiota, epithelial barriers, and inflammation could assume a key role in the carcinogenic process (77–80).

**Figure 1.**
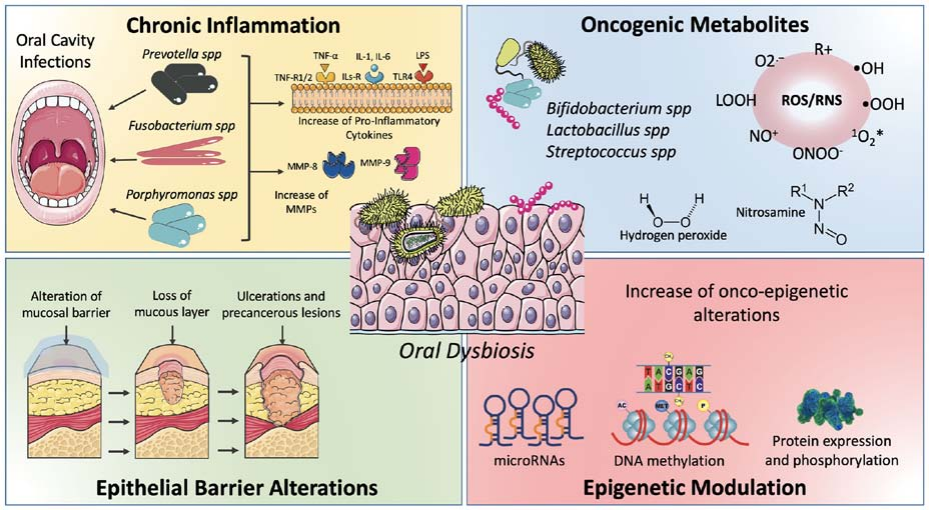
Oral microbiota dysbiosis is associated with oral cancer development through different mechanisms. Oral infections and dysbiosis are responsible for the instauration of a pro-inflammatory microenvironment of which inflammatory cytokines and matrix metalloproteinases favor the development and progression of tumors. Furthermore, the bacteria host in the oral cavity produces oxygen and nitrogen reactive species, as well as oncogenic metabolites (e.g., nitrosamines) to induce genetic damage to cells composing the oral mucosa. Another mechanism of neoplastic transformation mediated by oral dysbiosis is the alteration of the epithelial barriers predisposing the individuals for the development of chronic pre-cancerous lesions. Finally, oral dysbiosis is responsible for several epigenetic alterations predisposing the development of tumors (e.g., alteration of onco-miR or DNA methylation phenomena).

Finally, it was recently demonstrated that microbiota and oral mucosa dysbiosis lead to the accumulation of different epigenetic alterations predisposing for neoplastic transformation (Fig. 1) (81).

Chronic inflammation. According to data reported in the literature, approximately 25% of human cancer shares chronic inflammation as a risk factor, indicating that inflammation is one of the most important hallmarks of cancer (82). Several processes including cell proliferation, angiogenesis, mutagenesis and oncogene activation may be caused or facilitated by chronic inflammatory mediators that alter the normal homeostasis of cells and tissues (82).

Some anaerobic oral bacteria including *Fusobacterium, Porphyromonas* and *Prevotella* species are associated with periodontal diseases and lead to chronic inflammation. The inflammatory mediators secreted by bacterial cells are able to interact with the cells of different tissues inducing diffused inflammatory processes. Periodontal bacteria influence the paracrine production of different pro-inflammatory mediators, such as interleukins (IL-1, IL-6, IL-17, IL-23), tumor-necrosis factor-α (TNF-α), and proteinases able to deteriorate the extracellular matrix (MMP-8, −9 and −13) (83,84). The increased production of these inflammatory proteins is responsible for several types of cancer and the overexpression of these proteins (especially IL-6 and MMP-9) predict for a worse prognosis and an aggressive tumor phenotype (85–87).

The upregulation of cytokines and other inflammatory factors lead to the alteration of different molecular pathways, including metabolic pathways, responsible for the modulation of cell metabolism and proliferation. For example, RAGE protein expression changes significantly after periodontal diseases mediated by oral microbiota alterations, leading to carcinogenesis (88). Moreover, gram-negative bacteria release a pro-inflammatory lipopolysaccharide endotoxin (LPS) able to stimulate the production of IL1-β, IL-6 ant TNF-α by binding the TLR receptor of leucocytes (89,90). In particular, these inflammatory cytokines lead to the overexpression of other pro-inflammatory proteins stimulating the release of phospholipase A2, prostaglandins (PG) and acute phase proteins (91,92). Other studies demonstrated that high IL-1 levels favor a pro-angiogenetic microenvironment, supporting tumor spread (93–95). Simultaneously, IL-1 induces MMP-9, which has been associated with more aggressive phenotypes of carcinoma, higher invasiveness, and low patient survival (96,97).

Other studies demonstrated that tumor spread is also sustained by the overexpression of IL-6, which in turn leads to the upregulation of matrix-metalloproteinases, adhesion molecules and endothelial leukocyte adhesion molecules (98,99). All these data showed that interleukins, and in particular IL-6, are strictly involved in neoplastic transformation (100). Besides interleukins, altered levels of TNF-α, mediated by the alteration of Wnt and NF-κB pathways, were associated with the development of tumors (101,102). These data further corroborate the importance of NF-κB in cancer. Indeed, NF-κB acts as an immunostimulant factor against neoplastic cells; however, its protein expression is increased in several cancers acting as an oncogene (103,104).

The abovementioned evidence suggested that dysbiosis-associated cancer may rely on the abnormal activation of NF-κB (68). Thus, a fundamental role is played also by the immune system, which in the presence of pathogens stimulates the production of NF-κB (105).

Oncogenic substances production. Numerous substances produced by bacteria have been suggested to possess a carcinogenic action. Bacterial metabolism leads to the production of sulfur compounds, acids and free radicals, mainly nitric and oxygen reactive species, able to induce pro-tumoral genetic damage. Furthermore, several bacteria have an alcoholic metabolism responsible for the production of acetaldehyde, which sustains neoplastic transformation (90).

Regarding reactive oxygen species (ROS) and reactive nitrogen species (RNS), it is well established that alteration of NADPH oxidase and nitric oxide synthase (NOS) activity leads to the accumulation of these harmful substances which promote chronic inflammation and, as described in the above chapter, cancer development (106,107). Bacteria also play fundamental roles in these processes. Some peroxygenase oral microorganisms are involved in this process producing hydrogen peroxide (H_2_O_2_) and include *Bifidobacterium adolescentis, Lactobacillus acidophilus, L. fermentum, L. jensenii, L. minutus* (90,108), *Streptococcus gordonii, S. mitis, S. oligofermentans, S. oralis*, and *S. sanguinis* (109).

Other oncogenic substances produced by oral bacteria are represented by sulfides and nitrosamines. Bacteria including *Bacteroides* and *Firmicutes* species are capable to ferment the host excessive protein into sulfides and nitrosamines. These harmful substances are able to induce DNA damage in the oncogene or onco-suppressor genes (110,111).

In addition, oral microorganisms, including *Bifidobacterium, Lactobacillus, Lactococcus, Leuconostoc, Pediococcus, Peptostreptococcus stomatis* and *Streptococcus* produce several types of acids (lactic, acetic, butyric, isobutyric, isovaleric, and isocaproic acids), which reduce the environmental pH (112–114). These acids contribute to the establishment of an ideal tissue microenvironment favorable for cancer cell proliferation and metastatic spread (115,116).

Other studies highlighted the importance of superoxide dismutase (SOD) activity and its expression through the analysis of microbiomes detected in cancer samples and normal mucosa of oral cancer patients (117). The SOD activity is fundamental for inhibiting the detrimental effects of O_2_^.−^. In particular, it was shown that in tumor samples the presence of Fe^2+^ reacts with H_2_O_2_ leading to the production of harmful reactive species that promote neoplastic transformation by inducing DNA mutations affecting key genes involved in the regulation of cell cycle (118).

Yost *et al* reported that in cancer patients, both in tumor or normal sites, microbiome tryptophanase activities are a possible carcinogenetic mechanism. In particular, the higher metabolism of L-tryptophan to secondary metabolites (indole, pyruvate and ammonium) seems to be related to cancer development (117). In this context, the role of aryl hydrocarbon receptor (AHR) is fundamental (119). Similarly, other enzymes, such as glutamate dehydrogenase (GDH), are overexpressed in oral cancer patients and their imbalance may contribute to the alteration of the cellular redox state (120,121).

Finally, as stated above, several oral microorganisms (*S. gordonii, S. mitis, S. oralis, S. salivarius, S. sanguinis* (122), and *Candida* yeasts (123) are involved in alcohol metabolizing to acetaldehyde, which has a carcinogenic potential (68,123). All these bacteria constitute a risk for OSCC development due to their acetaldehyde production (124,125).

Integrity alteration of epithelial barrier. In a recent review, Pang *et al* described the role of dysbiosis in the alteration of epithelial barriers in homeostasis and immune activation, as well as the relationship with carcinogenesis (76). Changes taking place in anatomic structure or in microbial composition and mucus production may lead to an epithelial barrier dysfunction and microenvironment alterations (126). The consequent imbalance between epithelia/microbiota are key factors both in infections and other microbial diseases, including tumor (127,128).

Moreover, as aforementioned, pro-inflammatory conditions sustained by microbial alterations contribute to epithelial barrier alteration. According to Virchow (1881) (129), the inflammatory events are linked to microbiota and cancer. In addition, inflammation may modify the bacteria population, favor microbial translocation and induce the growth of specific bacteria (126,129,130).

Specific microbial metabolites (i.e., ROS and hydroxyl radical) and toxins [such as cytolethal distending toxin (CDT)] generate genomic damages inducing the neoplastic transformation of epithelial cells. Moreover, bacteria activate several signal transduction pathways through virulence genes, e.g., AvrA virulence factor. Another factor stimulated by bacteriocins and bacterial proteins is the transforming growth factor β (TGF-β), which induces abnormal cell proliferation (76,131).

Furthermore, TGF-β plays a role as an immunomodulating factor inhibiting dendritic cells (DCs); T-receptor cells thus act as tumor-promoter factors (132). Notably, the mechanism of TGF-β signaling in promoting tumorigenesis is also associated with the dysregulated inflammation microenvironment actuated by microbiota (76).

### 

#### Microbiota-induced epigenetic modulation

It has been widely demonstrated that environmental factors, including diet, lifestyle habits, and natural compounds, are responsible for both genetic and epigenetic alterations predisposing the development of several diseases, and have beneficial effects in preventing the development of chronic-degenerative disorders (133–140). In particular, dietary intake and food consumption modulate several cellular and molecular processes acting in a multifactorial manner (141–143). Indeed, the consumption of specific food and nutrients may modulate inflammatory and cell cycle regulatory pathways to maintain the optimal cell homeostasis, preventing the development of diseases (144,145). On the contrary, imbalances of nutrient consumption and/or absorption lead to epigenetic changes associated with certain pathologies, including cancer. The way by which food and nutrient intake is able to influence the onset of certain pathologies remains to be determined. However, findings have shown how foods can change the individual's redox state, the oral and gut microbiota, the DNA methylation status and the alteration of microRNA (miRNAs) expression levels (146–149).

In particular, the two latter epigenetic events are now been recognized as key mechanisms of neoplastic transformation and a plethora of diseases (12,87,150). In this context, recent studies have identified different miRNAs, a class of non-coding RNA of 20–22 nucleotides, associated with the development and progression of different tumors (151–157). In addition, numerous studies have demonstrated the presence of a dual relationship between microbiota and host microRNAs (miRNAs) and vice versa (158–160). Liu and co-workers showed that fecal miRNAs produced by epithelial cells and Hopx-positive cells were able to penetrate bacteria (*F. nucleatum* and *E coli*) modulating the gene expressions of bacteria and altering the microbiota composition and bacterial cell growth (161). These preliminary observations, obtained in Dicer1-deficient mice, allowed the researchers to conclude that fecal miRNAs exert an important role in the regulation of gut microbiota and microbiome suggesting their possible use as novel therapeutic strategies. Fecal miRNAs are not derived only from intestinal cells. Different studies demonstrated that fecal miRNAs can be derived from foods and can be absorbed by intestinal epithelia modulating the expression levels of host genes (162,163). These miRNAs are mainly planted exosome-derived miRNAs; however, several studies showed that milk-derived miRNAs play key roles (164–166).

All these food-derived miRNAs can presumably interact with oral and gut microbiota (167,168). On this basis, exogenous miRNAs may act as bacterial small RNA to interfere with bacterial gene expression modulating the entire microbiome (169,170). However, further studies are needed to deepen the knowledge on interactions between host-miRNAs and oral/gut microbiota. On the other hand, even the microbiota resident in the oral and intestinal mucosa may modulate the expression of specific miRNAs, thus highlighting a dual relationship between miRNAs and microbiota and their ability to influence each other (171).

One of the mechanisms by which microbiota alters the expression levels of host-epithelial miRNAs is the production of different metabolites leading to significant changes in host-cell metabolism resulting in the alteration of the gene and miRNA expressions (172–174). Pang *et al* investigated the association among dysbiosis, dysfunction of epithelial barrier and alteration of immune system to evaluate how dysbiosis stimulates carcinogenesis (76).

## 4. Oral bacteria with potential carcinogenetic activity

According to Zhao *et al* (175), oral cancer samples exhibited more bacterial species compared to healthy individuals. Specifically, *Catonella, Dialister, Filifactor, Fusobacterium, Parvimonas, Peptococcus* and *Peptostreptococcus* are the most overexpressed bacteria in OSCC samples with previous periodontitis. Notably, the evaluation of *Fusobacterium* bacteria was proposed as a diagnostic criterium for OSCC (175). In particular, *Fusobacterium nucleatum* is responsible, not only for opportunistic infections, but it was recently associated with several kind of cancers (175–179).

In addition, Yost *et al* (117) performed a preliminary meta-transcriptomic study in order to analyze the oral microbiome in oral squamous cell carcinoma patients and establish the connection with the molecular features of this tumor. Authors of that study found increased expression levels of *Fusobacteria* transcripts in both tumor and peritumoral tissue samples when compared to healthy individuals. Interestingly, they also showed that *Fusobacteria* virulence factors may be involved in the pathogenesis of oral cancer (117). Outcomes of that study are in agreement with findings of Nagy *et al* (50) who observed some oral bacteria correlated with keratinizing squamous cell carcinomas, including *Fusobacterium* sp. Moreover, Yang *et al* (180) correlated the microbiome variations to cancer progression. The *Fusobacteria* abundance was significantly higher in oral cancer patients showing progression and these data were more evident in stage 4 patients (7.92%) compared to healthy controls (2.98%) (180).

Lim *et al* (181) analyzed oral rinse to evaluate the microbiome variations and their correlation oral and head and neck cancers. Those authors identified a panel of bacteria (*Capnocytophaga, Corynebacterium, Haemophilus, Oribacterium, Paludibacter, Porphyromonas*, and *Rothia*) to discern oral cavity cancer patients (OCC), oropharyngeal cancer patients (OPC) and healthy subjects (181). On the basis of results obtained, the authors proposed the detection of these bacteria to predict the risk of OCC and OPC, reaching 100 and 90% sensitivity and specificity, respectively (182). The outcomes of that study are in agreement with previous studies (49,51,53,182–184) that indicated *Capnocytophaga gingivalis, Peptostreptococcus* sp., *Porphyromonas gingivalis, Prevotella* sp., and *Streptococcus* sp. as the oral microorganism mostly associated with OSCCs.

Yost and collaborators demonstrated that among various examined species, *Capnocytophaga gingivalis* was the more represented in non-tumoral sites. These contrasted results could be due to the different samples examined, as well as to the number of cases enrolled (117). Other bacteria associated with head, neck, and esophageal cancers were streptococci, of which *Streptococcus anginosus* represents the most important bacterium (51,185,186).

Recently, Yang *et al* (187) investigated the functional role of microbiota changes related to the genetic alterations observed in OSCC patients. The analysis of saliva samples demonstrated an imbalance in the oral cavity taxa showing a relative abundance of *Bacteroidetes* and *Firmicutes* in three different groups of oral cancer clustered according to the mutational status. Moreover, significant variations of microorganism diversity were highlighted through analysis of the three groups of patients. Based on these results, the authors proposed a possible association between mutation in OSCC and alteration of microbiota and microbiome (187). Differences in bacterial composition were also detected between precancerous lesions and cancer samples (53). The results of that study showed that salivary microbiota patterns were importantly modulated in the three analyzed groups. In particular, the genera *Bacillus, Enterococcus, Parvimonas, Peptostreptococcus*, and *Slackia* showed a predictive value for discrimination between precancerous and neoplastic lesions (53).

## 5. Anti-tumoral effects of probiotics

In recent years, it was widely demonstrated that the consumption of healthy foods enriched with probiotic acid lactic bacteria has positive effects regarding tumor prevention. Probiotics are able to reduce the mutagenic effects of harmful substances while modulating the expression of proteins involved in cell proliferation, apoptosis, inflammation, or immune system activation (188). Several *in vitro* experiments performed on cancer cells showed that probiotics possess anti-proliferative and pro-apoptotic effects in these tumor models (189). Lee and co-workers demonstrated that the cytoplasmic elements of *Bifidobacterium longum, L. acidophilus* and *L. casei* exert anti-neoplastic effects in different tumor *in vitro* models (190). Besides these probiotics, *Bacillus polyfermenticus* (191), *Lactobacillus acidophilus 606* (192), *LGG/Bb12* (193), *LGG/Bifidobacterium animalis subsp. lactis* (194), and *Lactobacillus rhamnosus GG* (9) possessed anti-neoplastic effects in colorectal cancer cell lines.

Few data are available on the effects of probiotics on oral cancer. In a recent study, HSC-3 OSCC cell lines were used to determine the effects of *Lactobacillus rhamnosus GG* (LGG) in increasing the antiblastic effects of geniposide, a derivate of *Gardenia jasminoides* which in preclinical studies showed important anticancer effects (58,195). The results obtained by the authors showed that the combined treatment with geniposide and LGG increased the apoptotic rate of HSC-3 cells. In particular, in a synergistic manner, LGG intensified the antineoplastic action of geniposide, supporting the tentative use of this combined therapy also in clinical practice. In another study, Asoudeh-Fard *et al* (196) demonstrated that *Lactobacillus plantarum* was able to inhibit and activate the MAPKs and PTEN pathways, respectively, playing a potential role in the regulation of cancer. Indeed, it is well known that PTEN and MAPKs are associated with the inhibition and the initiation of cancer development, respectively (196). Consequently, a possible use of *L. plantarum* for probiotics cancer therapy was proposed.

In addition, Aghazadeh *et al* (197) showed that *Acetobacter syzygii* strain secretions possess anticancer activity promoting the apoptosis induction in oral cancer cells. Interestingly, *Acetobacter syzygii* products were not involved in the alteration of homeostasis of the epithelial cell line (197). These findings seem to show the crucial role represented by the microorganism in controlling cancer development in oral tissues and encourage further investigations on the effect of probiotics on OSCC development.

## 6. Conclusions

This review widely discussed how the dysregulation of oral microbiota and oral mucosa homeostasis may represent modifiable risk factors associated with the development of OSCC. In addition, it was shown that certain bacterial strains may play a protective role against oral neoplastic transformation suggesting the possible use of probiotics administration as novel preventive and therapeutic strategies. In this scenario, several species have been strongly correlated with oral carcinoma, such as *Capnocytophaga gingivalis, Fusobacterium* sp., *Streptococcus* sp., *Peptostreptococcus* sp., *Porphyromonas gingivalis* and *Prevotella* sp., due to the fact that these bacteria may promote inflammation, cell proliferation and the production of some oncogenic substances (90). Recent findings have shown that the evaluation of oral microbiota and microbiome may provide important information on oral cancer oncogenesis, outcome prediction and therapeutic response (including immunotherapy) (198,199). In addition, thanks to the new high-throughput molecular technologies it was possible to define the precise composition and gene expression (microbiome) of oral bacteria in OSCC patients and normal controls identifying specific strains associated with an increased risk of OSCC development (1,200). Therefore, the analysis of circulating biomarkers (miRNAs, circulating DNA, specific proteins) represents a good approach for the assessment of oral cancer risk (201–203).

In this respect, the use of probiotics that in appropriate amounts give a health benefit to the host, including anti-tumoral effects, could be useful to promote cancer therapies representing a new step of the evolution of anticancer pharmacological treatments (190,204). Although *in vitro* and *in vivo* experiments demonstrated the beneficial effects probiotics (7–9), few data are available about the efficacy of probiotics in oral cancer. It is reasonable to hypothesize that the beneficial effects exert by probiotics in intestinal and colorectal cancer is similar to those acted in the oral mucosa. Starting from this assumption, future studies are required to explore the involvement of oral microbiota and its relationship with oral cancer.

## Figures and Tables

**Table I.** Most common microbial species represented in a normal bacterial flora of oral cavity.^a^

Bacterial species	Characteristics	Localization	Distribution
*Streptococcus mitis*	Gram-positive coccus,	Buccal surface	High
	facultative anaerobe.	Vestibule	High
	It has been associated with:	Tongue	High
	i) Bacterial endocarditis, especially in	Palate	High
	patients with prosthetic valves;	Tonsils	High
	ii) Infection in immunocompromised	Tooth surfaces	High
	patients, particularly immediately after tissue transplants and in neutropenic cancer patients.	Subgingival surface	High
*Streptococcus*	Gram-positive coccus,	Buccal surface	Medium
*sanguis*	facultative anaerobe.	Tongue lateral	Medium
	It has been associated with:	Palate	Medium
	i) Bacterial endocarditis, especially in	Tooth surfaces	High
	patients with prosthetic valves; ii) Infection in immunocompromised patients.	Subgingival surface	Medium
*Streptococcus*	Gram-positive coccus,	Buccal surface	Medium
*gordonii*	facultative anaerobe.	Vestibule	Medium
	It has been associated with:	Palate	Medium
	i) Bacterial endocarditis, especially in	Tooth surfaces	High
	patients with prosthetic valves; ii) Infection in immunocompromised patients.	Subgingival surface	Medium
*Gemella sanguinis*	Gram-positive coccus,	Buccal surface	High
	facultative anaerobe.	Vestibule	High
	It has been associated with:	Tongue lateral	High
	i) Bacterial endocarditis.	Palate	High
*Gemella*	Gram-positive coccus,	Tonsils	High
*haemolysans*	facultative anaerobe.	Tooth surfaces	High
	It has been associated with: i) Bacterial endocarditis.	Subgingival surface	Medium
*Granulicatella*	Gram-positive coccus,	Buccal surface	Medium
*elegans*	facultative anaerobe.	Vestibule	High
		Tongue lateral	Medium
	It has been associated with:	Hard palate	High
	i) Infective endocarditis.	Soft palate	Medium
		Tonsils	Medium
		Subgingival surface	Medium
*Granulicatella adiacens*	Gram-positive coccus, facultative anaerobe.	Buccal surface Vestibule	High Medium
		Tongue	High
	It has been associated with:	Hard palate	Medium
	i) Infective endocarditis.	Soft palate	High
		Tooth surfaces	High
		Subgingival surface	Medium
*Neisseria spp*.	Gram-negative diplococci, aerobic.	Buccal surface	Medium
		Tongue	Medium
	Most gonococcal infections are	Palate	High
	asymptomatic and self-resolving	Tonsils	Medium
	except for *N. meningitidis* and	Tooth surfaces	High
	*N. gonorrhoeae*.		
*Streptococcus mitis*	Gram-negative rod-shaped bacteria,	Buccal surface	Medium
	anaerobic.	Tongue	Medium
	It is a fundamental human pathogen	Soft palate	Medium
	in various anaerobic infections	Tonsils	Medium
	(i.e. transmissible subcutaneous	Tooth surfaces	Medium
	infections).	Subgingival surface	Medium

a Microbial species as indicated (205).

**Table II.** Predominant microbial communities associated with OSCC.

Bacterial species	Localization (206)	Refs.	Type of sample
*Actinomyces spp*	Tooth surface	(52)	Saliva samples
*Bacteroides spp*	Gingival crevice	(110,111,187)	Saliva samples
*Bifidobacterium spp*	Tooth surface	(112,113)	Plaque biofilm samples
*Capnocytophaga spp*	Gingival crevice Tongue	(49)	Samples of gingival SCC and normal gingiva
		(50)	Oral rinse
		(51)	Saliva samples
		(53)	Oral swabs
		(117)	Oral rinse
		(181–184)	Samples of oral tumour and precancerous leukoplakia Samples of tongue and floor SCC and normal tissues
*Catonella spp*	Gingival crevice	(175)	Oral swabs
*Clostridium spp*	Gingival crevice	(52)	Saliva samples
*Dialister spp*	Gingival crevice	(175)	Oral swabs
*Enterobacteriaceae spp*	Gingival crevice	(52)	Saliva samples
*Enterococcus spp*	Tongue, tooth surface	(53)	Saliva samples
*Filifactor spp*	Gingival crevice	(175)	Oral swabs
*Firmicutes spp*	Gingival crevice	(187)	Saliva samples
*Fusobacteria spp*	Gingival crevice	(51)	Saliva samples
		(117)	Oral swabs
		(175)	OSCC biopsies and deep-
		(176)	epithelium swabs
		(180)	Oral rinse
*Haemophilus spp*	Oropharynx	(52)	Saliva samples
	Tonsil	(181)	Oral rinse
*Lactobacillus spp*	Tooth surface	(112,113)	Plaque biofilm samples
*Lactococcus spp*	Tooth surface	(112,113)	Plaque biofilm samples
*Leuconostoc spp*	Tooth surface	(112,113)	Plaque biofilm samples
*Oribacterium spp*	Gingival crevice Tooth surface	(181)	Oral rinse
*Paludibacter spp*	Gingival crevice Tooth surface	(181)	Oral rinse
*Parvimonas spp*	Gingival crevice	(53)	Saliva samples
	Tooth surface	(175)	Oral swabs
*Pediococcus spp*	Tooth surface	(112,113)	Plaque biofilm samples
Peptococcus spp	Gingival crevice	(175)	Oral swabs
*Peptostreptococcus spp*	Tooth surface	(51)	Saliva samples
	Gingival crevice	(53)	Samples of dental abscess,
		(90)	endodontic or pericoronal
		(114)	infection, periodontal pocket
		(175)	Oral swabs
		(183)	Samples of tongue and floor
		(184)	SCC and normal tissues
*Porphyromonas gingivalis*	Tongue	(47–49)	Samples of gingival SCC
	Gingival crevice	(51,53)	and normal gingiva
		(90)	Saliva samples
		(183)	Samples of tongue and floor
		(184)	SCC and normal tissues
*Prevotella intermedia*	Gingival crevice	(50,51)	Oral rinse
		(90)	Saliva samples
*Rothia spp*	Gingival crevice	(122)	Oral rinse
	Tooth surface		
*Slackia spp*	Gingival crevice	(53)	Saliva samples
*Streptococcus spp*	Gingival crevice	(50)	Oral rinse
	Oropharynx	(51)	Saliva samples
	Tooth surface	(52)	Samples of oral tumour and
	Tonsil	(90)	precancerous leukoplakia
	Gingival crevice	(122)	
	Oropharynx	(124)	
	Tooth Surface	(125)	
	Tonsil	(182)	

## Data Availability

The datasets used and/or analyzed during the current study are available from the corresponding author on reasonable request.
